# Several machine learning techniques comparison for the prediction of the uniaxial compressive strength of carbonate rocks

**DOI:** 10.1038/s41598-022-25633-0

**Published:** 2022-12-05

**Authors:** Mohamed Yusuf Hassan, Hasan Arman

**Affiliations:** 1 Department of Statistics, College of Business, United Arab Emirates University, P.O. Box: 15551, Al Ain, United Arab Emirates; 2 Department of Geosciences, College of Science, United Arab Emirates University, P.O. Box: 15551, Al Ain, United Arab Emirates

**Keywords:** Civil engineering, Environmental sciences

## Abstract

In engineering practices, it is critical and necessary to either measure or estimate the uniaxial compressive strength (UCS) of the rock. Measuring the UCS of rocks requires comprehensive studies in the field and in the laboratory for the rock block sampling, coring, and testing. These studies are time-consuming, expensive and go through difficult processes. Alternatively, the UCS can either be estimated by empirical relationships or predictive models with various measured mechanical and physical parameters of the rocks. Previous studies used different methods to predict UCS, including least squares regression techniques (MLR), adaptive neuro-fuzzy inference system (ANFIS), Sequential artificial neuron networks (SANN), etc. This study is intended to estimate the UCS of the carbonate rock by using a simple, measured Schmidt Hammer (SHV_C_) test on core sample and a unit weight (γ_n_) of carbonate rock. Principal components regression (PCR), MLR, SANN, and ANFIS are employed to predict the UCS. We are not aware of any study compared the performances of these methods for the prediction of the UCS values. Based on the root mean square error, mean absolute error and R^2^, the Sequential artificial neural network has a slight advantage against the other three models.

## Introduction

Carbonate rocks are the most common rock types in engineering structure foundation levels, and they have various uses in the construction industry. The short and the long-term stability and the performance of structures are usually determined by the uniaxial compressive strength (UCS) test. Designation of the UCS of rocks in the field and in the laboratory need fundamental responsibility in the civil, geological, mining, and rock engineering applications for the design of structures either on or inside rock materials.

The UCS test is standardized by the American Society for Testing and Materials^1^ and the International Society for Rock Mechanics^2^, and it is widely accepted test used to attain the strength of rock materials. Many researchers have been questioning its destructive nature and the potential complications involved in handling, preparing, and testing the samples, particularly in weak rocks. Nevertheless, even for hard rocks like carbonates (limestone), the UCS test is still very difficult, expensive, tedious, and time-consuming, and it requires to test a number of well-prepared rock samples in order to obtain consistent strength value for the rock material^3–8^. Frequently, nondestructive indirect methods like Schmidt hammer, Shore hardness, slake durability, P-wave velocity, point load index test, etc. are used to predict the UCS of the rocks in the field and in the laboratory. These methods have been continuously used as an alternative method since they are simple, more practical and do not require complicated procedures on the testing equipment. Besides, these methods are faster and more economical compared to the traditionally-suggested UCS testing methods^6,9–19^.

In recent years, various modelling techniques, such as simple and multivariable regression analyses, fuzzy inference system, neural network, other machine-learning algorithms, etc., have gained more attention and perceived as the best models to be used the prediction of the strength of rock materials. A reliable database for the mechanical and the physical rock properties is critical for the predictive models to assess the UCS of the rock^3,13,20–37^.

Many researchers have suggested different prediction techniques to estimate the strength of various rock types. Alvarez Grima and Babuska^20^ presented fuzzy modelling application to predict the UCS of several rock types from a data file, which contained 226 rock samples. They showed that the fuzzy model was not only accurate enough, but also provided insights about the nonlinear relationships among the measured variables. Kahraman^3^ reported linear and non-linear correlations among different rock parameters, such as point load, Schmidt hammer, etc., from 48 rock types. He indicated that all empirical methods studied could be used to predict the UCS of the rock except the impact strength test values. Gokceoglu^21^ stated that it would be possible to predict the UCS of the Ankara agglomerates from their petrographic composition with the fuzzy triangular chart, which exhibited high precision prediction compared to the variance accounts for (VAF), and the root mean square error (RMSE) measures. Basu and Aydin^13^ predicted the UCS using 40 granitic air-dried core specimens by point load test. They critically emphasized how cone penetration of the point load test depended on rock type and its microstructure. Then, they suggested that the related standards should be approved accordingly. Karakus and Tutmez^22^ developed fuzzy and multiple regression models to predict the UCS of intact rocks. They compared two models and showed that the best prediction model was the fuzzy model. Kilinc and Teymen^23^ determined mechanical properties of 19 different rock types using regression analyses. They found reliable correlations among various parameters and showed that the strength of rocks could be predicted using simple and non-destructive methods. Gokceoglu et al.^24^ suggested some predictive models to predict the UCS of some clay-bearing rocks based on their slake durability indices and clay contents. They calculated statistical performance accuracy measures to compare the predictive performance of the models and reported that the fuzzy inference system had a better prediction capacity compared to the regression models. Yurdakul et al.^25^ introduced an artificial neural networks (ANN) predictive model to assess the UCS of 37 different carbonate rocks from Schmidt hardness values. Their study revealed that the ANN-based model provided better results compared to the various regression models. Yagiz et al.^26^ assessed the influence of slake durability cycles on the prediction of UCS and modulus of elasticity for 7 types of carbonate rocks using artificial neural networks and non-linear regression techniques. They reported that new performance index (PI) and degree of consistency (Cd) could be accepted as indicators for assessing the accuracy of complex models, and they have indicated that ANN models provided better estimation for the rock properties than the regression techniques. Misra and Basu^27^ presented comparative studies of estimating the UCS of 3 different rock types with fuzzy inference system and regression analysis using some index properties of rocks. Their study showed that fuzzy inference system and multiple regression analyses were better than simple regression analyses in predicting the UCS of rocks, but one should be careful about plausible error in multiple regression analysis. Ceryan et al.^28^ established predictive models to estimate the UCS of various carbonate rocks by artificial neural networks. They concluded that the Levenberg–Marquardt algorithm based on ANN (LM-ANN) was capable enough to predict the UCS of the rocks, and they suggested to use it the estimation of the other index parameters. Yesiloglu et al.^29^ investigated the possibility of estimating the UCS of granitic rocks from their mineral contents by using adaptive neuro fuzzy inference system (UNFIS). Their analyses revealed that the chosen non-linear multiple regression and ANFIS models could be employed for the estimation of the UCS of the granitic rocks, and the ANFIS model outperformed the non-linear multiple regression model. Dindarloo and Siami-Irdemoosa^30^ reported in their studies about the estimating of 117 UCS representative core specimens of carbonate rocks by gene expression programming (GEP). The GEP performed better than ANNs in predicting the UCS of the carbonate rocks. Gul et al.^31^ employed ANN and multiple regression models to estimate the UCS strength of different rocks, and their study showed that the performance of ANN was better than that of the regression model, particularly the estimation of the strength and the modulus parameters of the rocks. Madhubabu et al.^32^ predicted the UCS and modulus of elasticity of carbonate rocks using multiple linear regression analysis (MLRA) and ANN. They used 163 sample data from previous researchers in addition to their own data for the study, and their results showed that ANN performed better than the MLRA. Aboutaleb et al.^33^ studied to estimate the UCS and elastic modulus of 482 carbonate core samples using simple and multiple regression analysis (SR and MRA), ANN and support vector regression (SVR). They found that the SVR model was desirable; it has provided competitive advantage against other methods due to its running time. Hassanv et al.^34^ used various models such as multiple linear regression (MLR) and ANN to estimate the UCS of carbonate oil reservoir by utilizing 121 core datasets from one of the Iranian oil field carbonates. They reported that MLR provided best results compared to other models with respect to correlation coefficient values. Wang et al.^35^ considered the random forest predictive model to estimate the UCS of rocks by utilizing data collected from previous research and using simple index parameters. They concluded that the predictive results aligned with laboratory tests could be used in the fields of rock mechanics and engineering geology to predict the UCS values. Saldana et al.^36^ used statistical analysis and machine learning models to predict the UCS of travertine. They reported that multiple regression model with the explanatory variables such as porosity and P-wave velocity was the best model to estimate the UCS of travertine. Yang et al.^37^ foreseen the rate-dependent compressive strength of rocks utilizing 164 experimental datasets by employing four machine learning models; extreme learning machine (ELM), random forest (RF), SVR and hybrid model of particular swarm optimization (PSO)-SVR. Their study showed that the PSO-SVR model provided a higher accuracy of prediction with a less prediction error compared to the other three models.

In previous studies, the empirical or predictive models such as least squares regression techniques, adaptive neuro-fuzzy inference system, artificial neuron networks and others with different measured mechanical and physical parameters of the rocks has been used to predict UCS. However, there is no study compared the performance of these methods for the prediction of the UCS values based on the available literature. Nevertheless, this study intends to develop the best predictive models of principal components regression, multiple regression, sequential artificial neural networks and adaptive neuro-fuzzy inference system to estimate the UCS of the carbonate rocks from their simple measured parameters of the SHV_C_ and γ_n_, and to compare the performances of these methods for the prediction of the UCS values. Such an approach, especially during a preliminary design stage of any engineering structures, could be faster and economical if different laboratory test results indicate variations, and it is difficult to adopt any conventional statistical approaches. Therefore, machine-learning techniques would provide better estimation of the UCS of the carbonate rocks. On the other hand, machine-learning techniques are powerful in dealing with non-linear systems, but they need large enough data set that can represent the system to be investigated.

## Sampling and experimental studies

**Figure 1:**
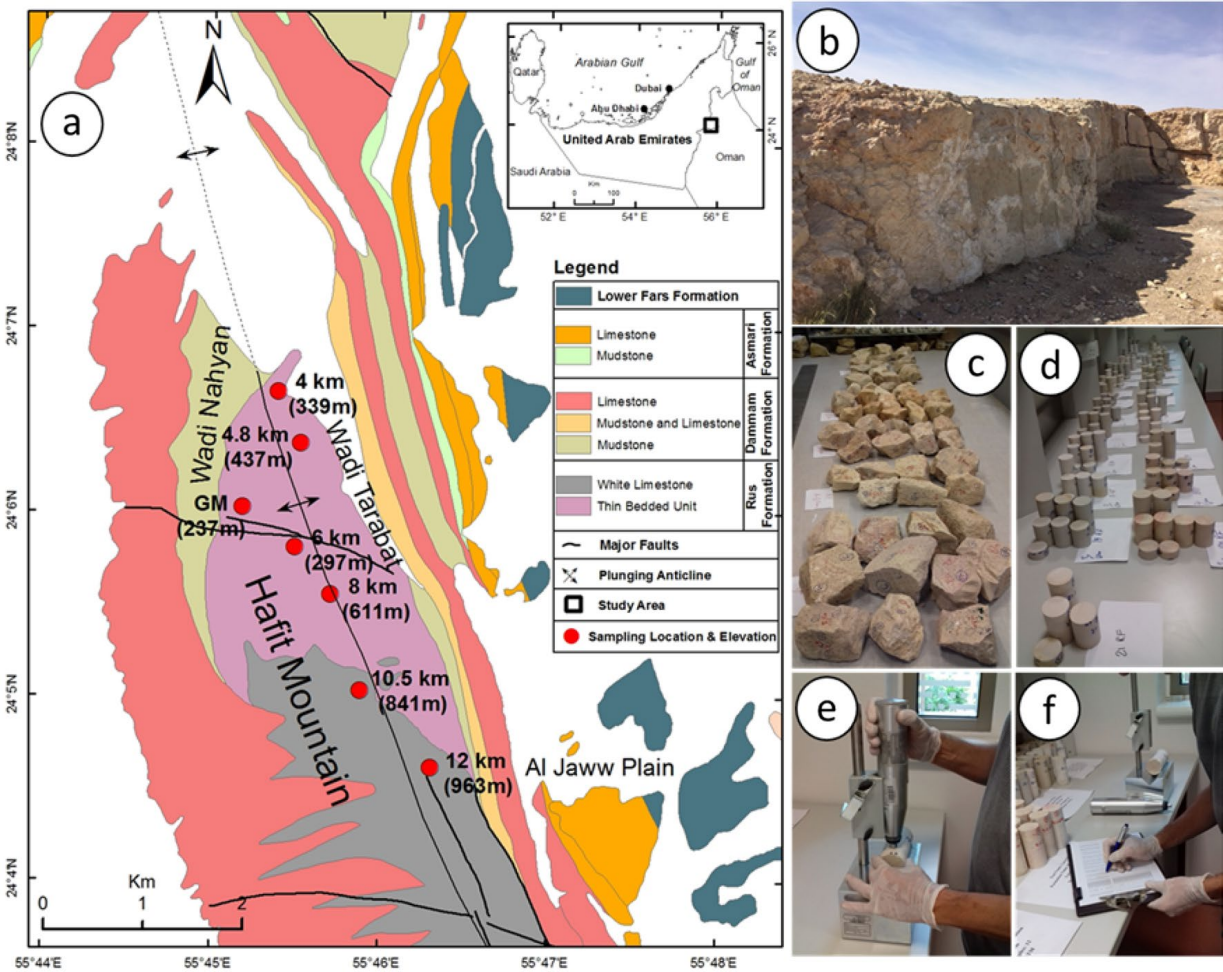
(**a**) Geological map of the Haft Mountain and sampling location with elevation from a.s.l. (generated with ArcGIS 10.8^40^), (**b**) outcrop of limestone, (**c**) rock block samples, (**d**) core samples, (**e**,**f**) SHV_C_ test on core sample.

To prepare test samples for various tests, more than 100 rock blocks were collected from the study area^38,39^, which was about 12 km long including seven targeted locations as sampling points (Fig. 1a,b). Each rock block, which was brought to laboratory, was carefully inspected to make sure that the rock blocks were free of any visible-macro defects such as alteration zones, cracks and fractures, so it has to provide standard test specimens (Fig. 1c). Test samples for physical and mechanical tests were prepared from 94 selected rock blocks following the suggested ASTM and ISRM standards and all tests were conducted on intact rock samples according to suggested tests standards, including the uniaxial compressive strength^1^ (Fig. 1d–f). If the tests did not meet the suggested standards due to either core sample features or rock failing unexpectedly along the existing invisible weakness plane, their results were not used for the analyses.

## Data quantitative and qualitative assessments

**Figure 2:**
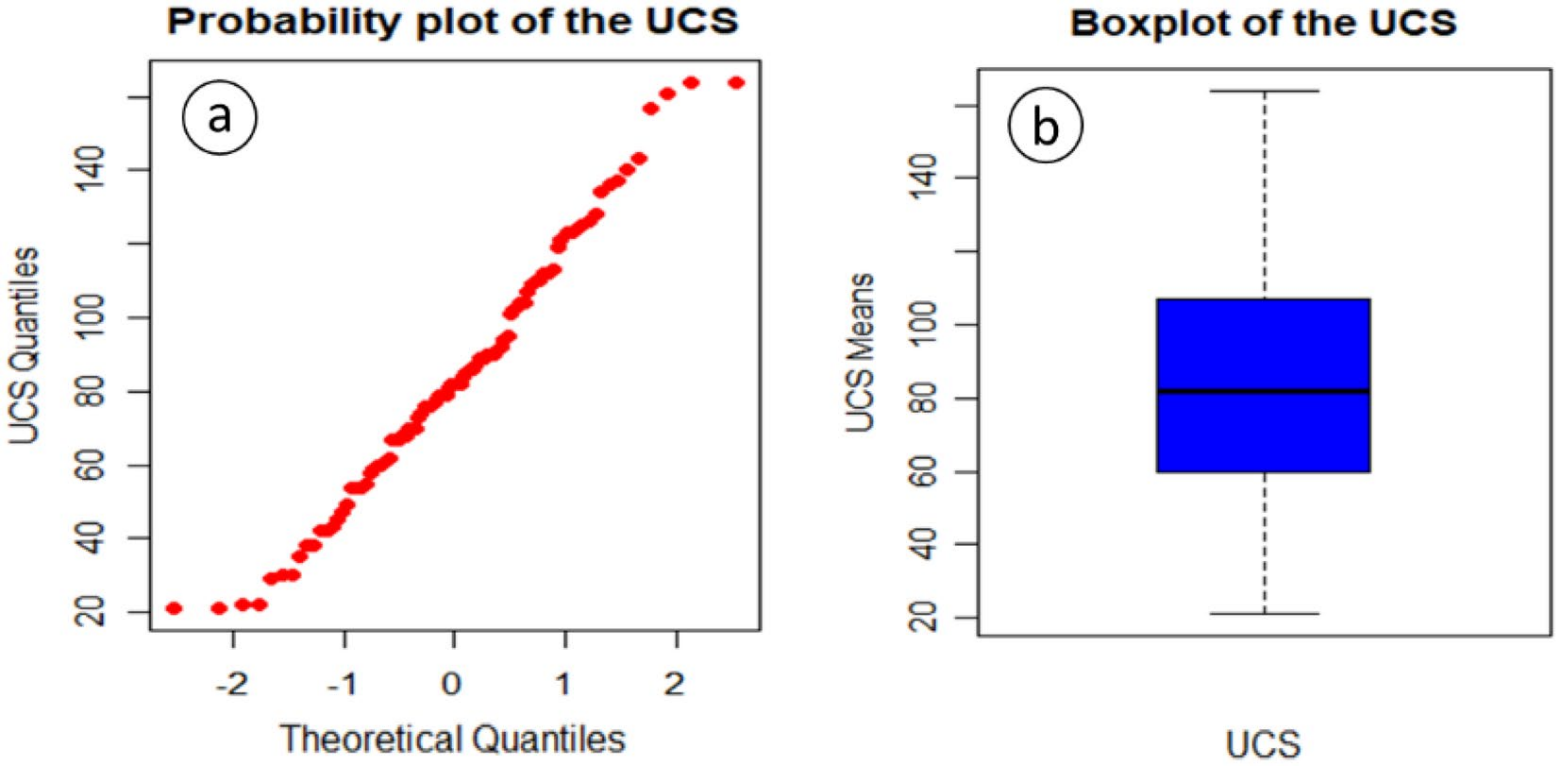
(**a**) Normal probability of the UCS, (**b**) boxplot of the UCS.

After the data were collected, qualitative and quantitative assessments were conducted. Figure 2a displays the normal probability plot and Fig. 2b displays the boxplot of the UCS values. Both plots show that there are no outliers and the UCS distribution is relatively symmetric. Besides, a Shapiro–Wilk normality test has resulted (p value > 0.1), which is an indicator that there is no departure of normality. Descriptive statistics of all the variables is summarized in Table 1, including 95% confidence intervals for the means of the three variables.

**Table 1:** Descriptive statistics of the data.

Variable	N	Mean	StDev	SE mean	95% CI for μ
UCS	93	83.85	34.32	3.56	(76.78, 90.92)
SHVc	93	34.15	4.13	0.43	(33.30, 35.00)
γ_n_	93	23.99	1.188	0.12	(23.75, 24.24)

**Figure 3:**
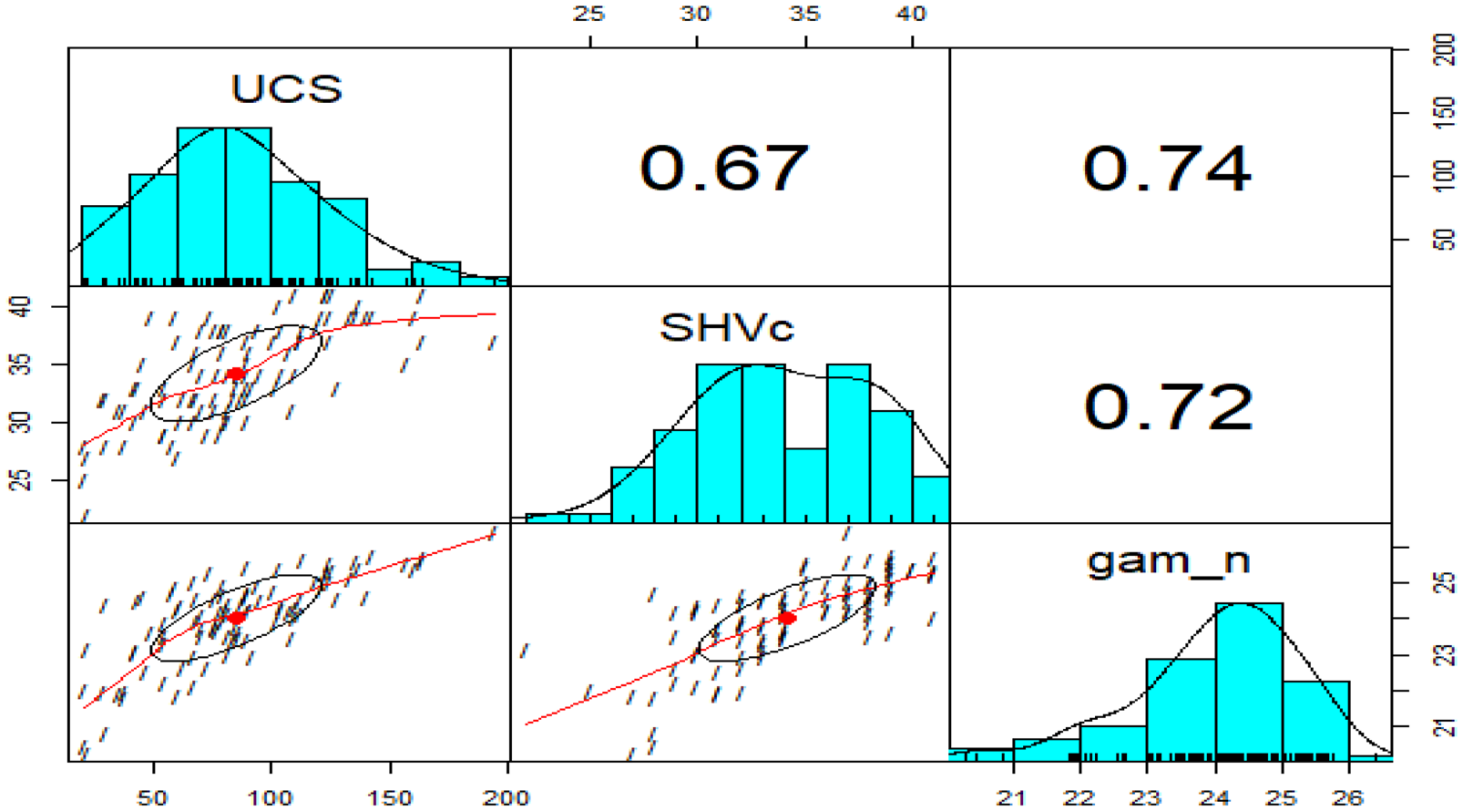
Pairwise scatterplots of the three variables.

Figure 3 presented pairwise scatterplots, empirical density plots, and the correlations among the different variables. It can be seen from the scatterplots that UCS is linearly related to each of the predictor variables, and the density plots show that SHVc is bimodal with modes at 32 and 29, while all the other variables are unimodal. In addition, Fig. 3 shows that the UCS has highly significant correlations with all the explanatory variables.

## Model development

This study compares the performances of principal components regression (PCR), sequential artificial neural networks (SANN), multiple linear regression (MLR) and the adaptive neuro-fuzzy inference system (ANFIS) in Predicting UCS values. These four soft computing techniques have a wide range application in many different disciplines. So far, we are not aware any applications of comparing these machine learning techniques in the geology literature, and it seems that this study is the first to tackle this problem.

### Principal components regression (PCR)

Principal components analysis (PCA) is an unsupervised technique used to reduce the dimension of the datasets via singular value decomposition. It was first introduced by Karl Pearson^41^, and later independently developed by Hotelling^42,43^. Jolliffe^44,45^ considered different forms of the procedure, and Jeffers^46^ and Chattopadhyay^47^ investigated several case studies of its applications. The PCA procedure finds an orthogonal set of linear combinations of the variables in an ***n*** × ***m*** data set **X** via a singular value decomposition. Suppose U **Σ V**^**T**^ is the singular value decomposition of **X**, where **k-th** principal component of **X** is denoted by ***z***_***k***_ = **Xv**_**k**_, then **Z**_**k**_ is the matrix of the first **k** principal components, i.e.** Zk = XV**_**k**_ where **V**_**k**_ contains the first **k** right singular vectors as columns of the matrix **V = [V**_**1**_**, …, V**_**n**_**]**, which is a matrix of size ***nxn*** whose columns are the normalized eigenvectors of **X**^**T**^**X**. PCR utilizes the resulted components of the data matrix XX, by regressing the response **Y** onto **Z**_**k**_, that is, it fits the model **Y = βZ**_**k**_** + ε**. If the principal components are chosen correctly, this regression can overcome multicollinearity and lead to high accuracy prediction results^48^.

### Sequential artificial neural network (SANN)

Sequential artificial neural network (SANN) is one of the most powerful supervised machine-learning algorithms. This computational algorithm has been employed to the prediction of different problems with different structures and variations for the last few decades^31,49^. SANN contains three different layers: input layer, hidden layers, and an output layer. The prediction performance of the algorithm on a given problem depends on its structure^50^, which is the choice of the number of hidden layers and how many neurons for each layer. There are number of available programming languages used to implement the algorithm, but the most used are: python and R. Some of the packages for these languages include deepnet, neuralnet, mxnet, h2o, keras, and tensorflow. In this study, three of the most used packages, namely, PLS, Keras on TensorFlow and Frbs.learn were employed.

### Adaptive neuro fuzzy inference system (ANFIS)

**Figure 4:**
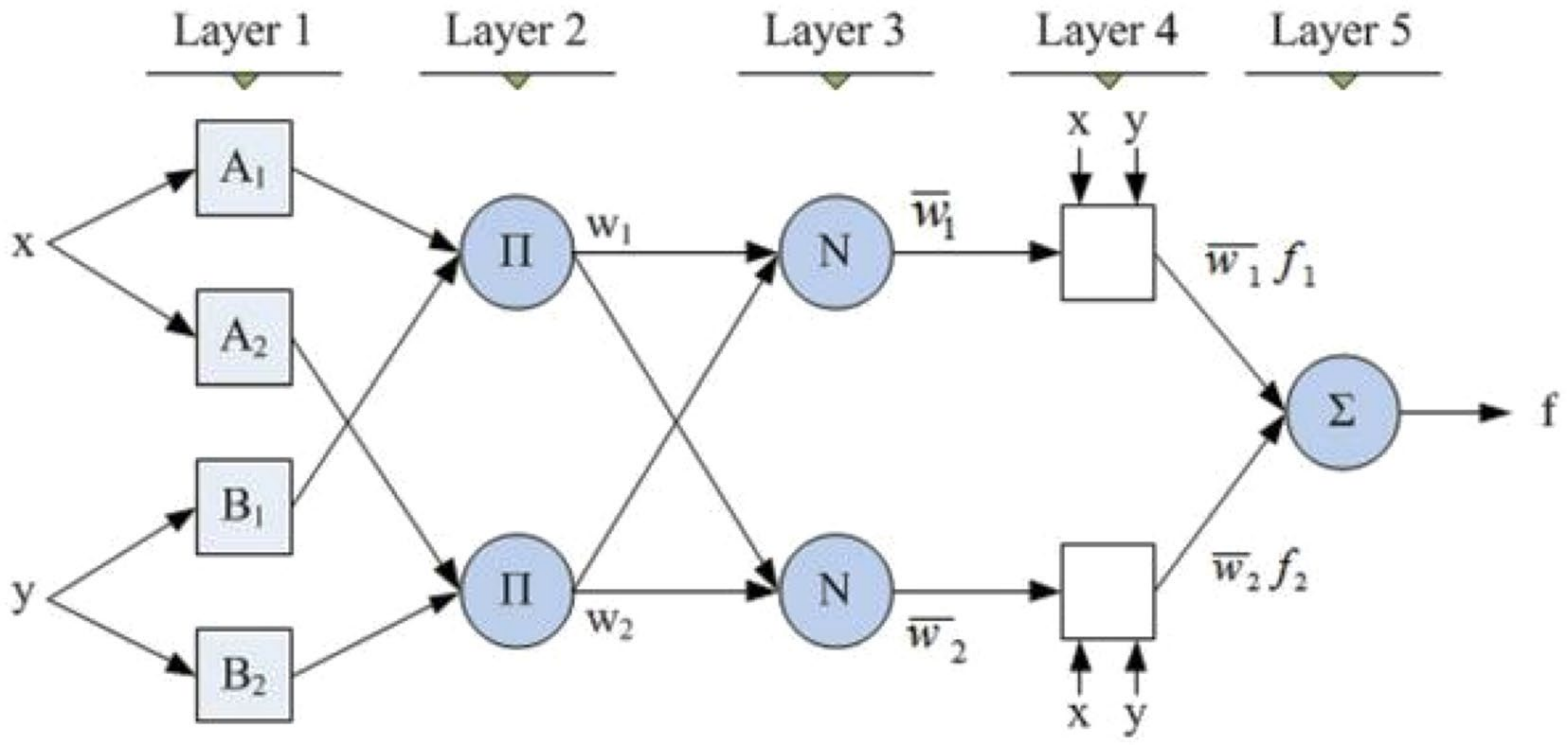
ANFIS network architecture.

ANFIS is a combination of artificial neural networks and fuzzy inference system. Fuzzy inference is the process of formulating the mapping from a given input to an output using fuzzy logic, Fuzzy logic was developed to address these issues, and it was first introduced by Zadeh^51^. These systems have membership functions that characterizes their fuzzy sets. There are two main types of fuzzy inference system, Mamdani-type^52^ and Sugeno-type^53^. The main difference between the two techniques is that the output of the Mamdani-type membership functions are fuzzy sets whereas those for Sugeno-type output membership functions are either linear or constant^54,55^. In this study, a Sugeno-type model is employed to implement the ANFIS algorithm. Below is presented the two IF–THEN rules of the first order ANFIS model, and its architecture is displayed in Fig. 4.

Rule 1: IF x is A_1_ AND is y B_1_, THEN, f_1_ = p_1_x + q_1_y + r_1_,

Rule 2: IF x is A_2_ AND is y B_2_, THEN, f_2_ = p_2_x + q_2_y + r_2_.

Where x any y are the inputs, and p_i,_ q_i_ and r_i_ are the parameters of the model.

The ANFIS structure has five layers^56^, namely, fuzzy layer, product layer, normalized layer, de-fuzzy layer, and output layer as shown in Fig. 4.

Fuzzifying Layer 1: Every node in this layer is an adaptive node, where the output of the layer is defined as follows:

1 $${out}_{1,i}={\mu }_{Ai}\left(x\right) i=\mathrm{1,2}.$$

2 $${out}_{1,i}={\mu }_{Bi-2}\left(y\right) i=\mathrm{3,4}.$$

where ${\mu }_{Ai}\left(x\right)$ and ${\mu }_{Bi-2}\left(y\right)$ are the membership functions.

Product Layer 2: The output of each node in this layer w_i_ represents the firing strength of a rule.

3 $${out}_{2,i}={\mathrm{wi}=\mu }_{Ai}\left(x\right) \mathrm{x }{\mu }_{Bi}\left(y\right), i=\mathrm{1,2}.$$

Normalized Layer 3: The output signal of the ith node is calculated by the ratio of the ith rule’s firing strength to the sum of the firing strengths for all rules as follows:

4 $${out}_{3,i}=\frac{{w}_{i}}{{w}_{1}+{w}_{2}}$$

Defuzzifying Layer 4: Every node in this layer is an adaptive node with a node function containing the resulting parameters from the normalized firing as follows:

5 $${out}_{4,i}={{f}_{i}=\overline{w} }_{i}({p}_{i}x+{q}_{i}y+{r}_{i})$$

Output Layer 5: This last layer contains a single fixed node labeled, which adds all the input signals to calculate the total final output as follows

6 $${out}_{5,i}=\frac{{\Sigma }_{i}{w}_{i}{f}_{i}}{{\Sigma }_{i}{w}_{i}}$$

## Results and discussion

In this study, PCR, MLR, SANN and ANFIS are used for the modeling of the test samples for the physical and the mechanical test results from a selected 93 rock blocks. Two independent variables, SHVc, and γ_n_, are chosen to predict the UCS values. After the models are trained, the performances of the four models are compared using the results of the accuracy measures, coefficient of determination (R^2^), root mean square error (RMSE) and mean absolute Error (MAE) to determine the best model in predicting UCS. First, the data were randomly split into training and test sets with an 80:20 ratio (80% training and 20% testing^57^, and then the independent variables of the training data were standardized by changing into z-scores. The normalization method is widely used to improve the convergence of the machine-learning algorithms^58,59^. After data normalization, a ten-fold cross-validation (CV), which is a resampling method used to validate the performance of a fitted model, is used to choose the best model. The data are divided into 10 subsamples. (9/10 proportion of the data is used to build the model, and the remaining 1/10 proportion is used as a test; this procedure is repeated 10 times.

### Sequential artificial neural network (SANN)

**Figure 5:**
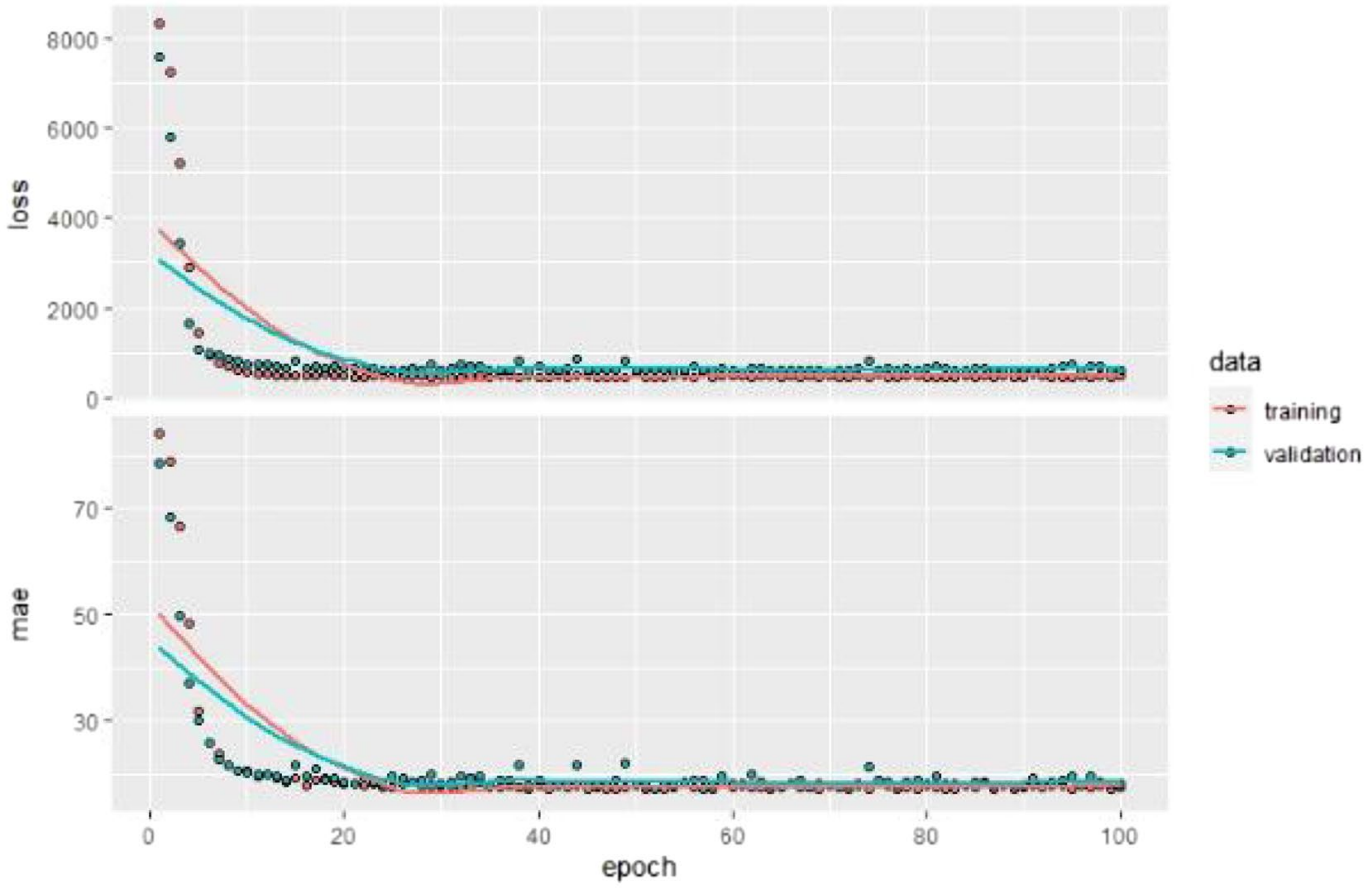
MSE and MAE as a function of the number of epochs.

Keras on TensorFlow in the R package is used to build the SANN model. They are set up to be used in an R environment. Keras and Tensorflow are powerful packages that are accessed through python. TensorFlow is an open-source platform for machine learning while Keras is a high-level neural network application-programming interface (API) written in Python on the top of TensorFlow. Both Keras and TensorFlow are used for the development and implementation of deep-learning models. A Keras sequential model with the rectified linear unit (Relu) activation function is used to identify the best model for predicting UCS. The training parameters of the SANN model include, the loss function (MSE), epochs—which is the number of times the dataset is passed through the network, batch size, and the learning rate. In this study, the best SANN model identified by the accuracy measurements has two hidden dimensions with four and three neurons, respectively. Details about the number of hidden layers and their neurons that are needed for any given set of input layers can be found at Lippmann^60^ and Bishop^61^. The chosen model has a learning rate of 0.05%, 100 epochs, batch size of 16, and a validation split of 0.20. The number of the parameters for this model are 31, 15 for the first hidden layer, 12 for the second hidden layer, and 4 for the output layer. The model is trained very well with the data, and the training error rate decreased very sharply, as can be seen in Fig. 5; both the MSE and the MAE decreased very fast before 25 epochs and stabilized thereafter. The coefficient of determination (R^2^) for the SANN model is 45%.

### Principal components regression (PCR)

**Figure 6:**
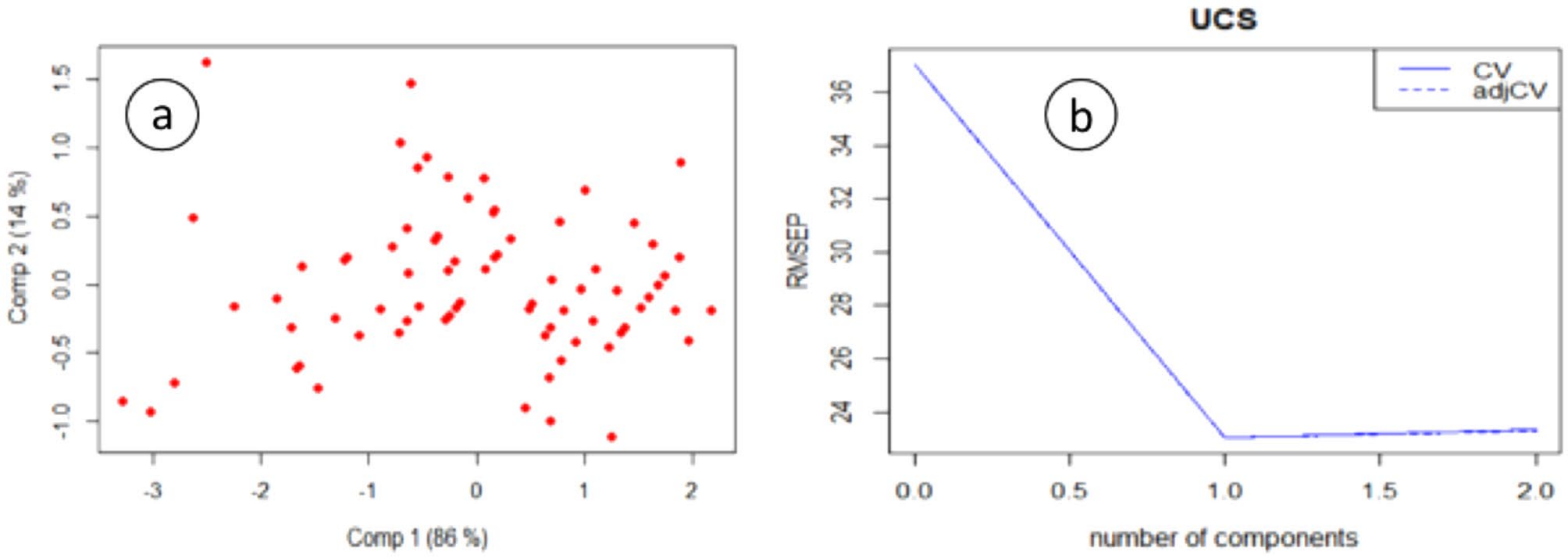
(**a**) Scatter plot of the components, (**b**) root mean square error plot.

The PCR model is fitted to the training data set, and the best model identified by cross-validation has two components. The first component explains 86% of the variability in the original dataset while the second component explains 14%, see for example Fig. 6a.

The ten-fold cross-validation errors are computed for each of all the possible components, and their corresponding root mean squared errors for prediction (MSEP), cross-validation (CV) and the adjusted cross-validation (adjCV), CV is a bias-corrected CV. Mevik and Cederkvist^62^ estimates are reported, and both are decreasing very fast as can be seen in Fig. 6b. The smallest cross-validation error is attained when the number of the components are 2. However, from the plot, one can see that the cross-validation error is almost the same even if only one component is chosen for the model. This indicates that a model that has only component is enough for the prediction of the UCS values.

### Regression

The most used feature selection methods to identify the best regression model are the forward selection, backward elimination, and the best subsets. The best fitted regression model identified for the prediction of the UCS, using the training data, is the model with the two explanatory variables, SHVc and γ_n_. All the parameters were highly significant (see Table 2), and the variance inflation factor (VIF) is very low (2.03) indicating that multicollinearity is not detected. VIF values more than 10 are considered to indicate serious multicollinearity.

**Table 2:** MLR parameter estimates.

Variable	Coeff	T-Value	p value
Constant	− 406	− 7.53	**< 0.001**
SHVc	2.398	2.67	**< 0.001**
γ_n_	17.08	5.75	**< 0.001**

Significant values are in [bold].

**Figure 7:**
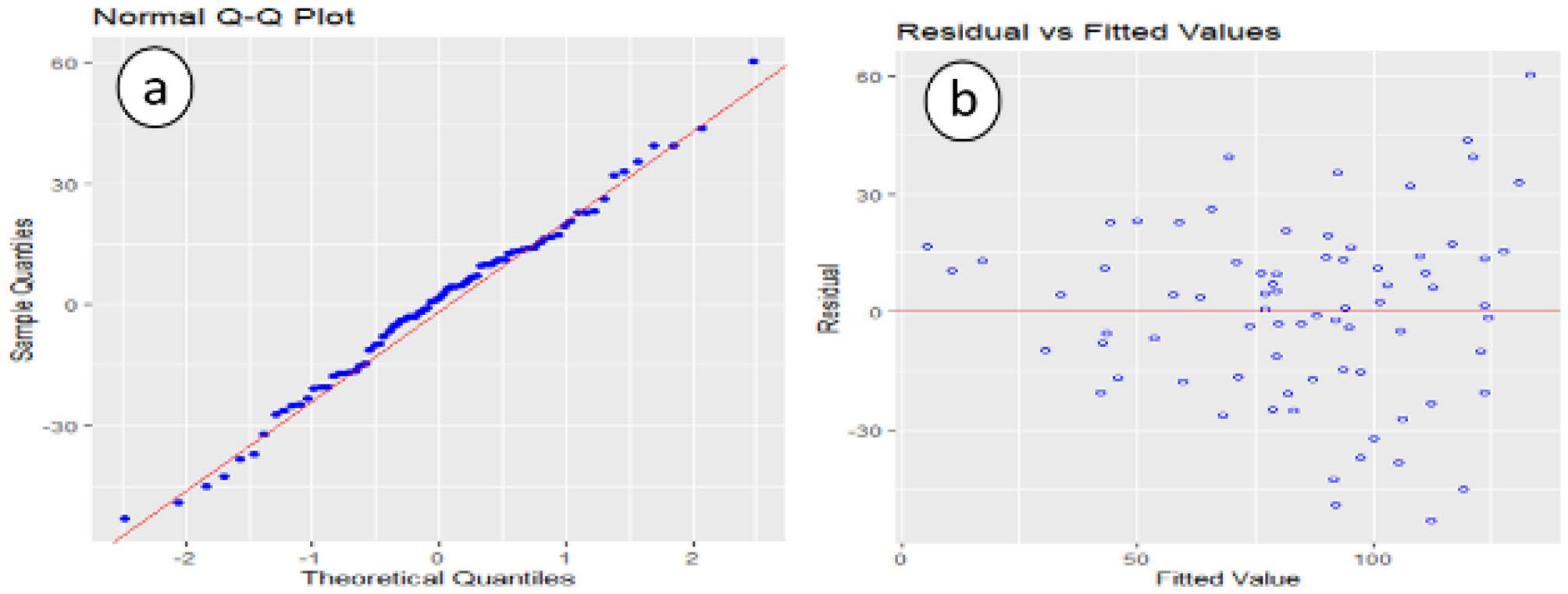
(**a**) Residuals normality plot, (**b**) residuals equality of variances plot.

Kolmogorov–Smirnov test is used to test the normality assumption of the residuals, and a p value of more than 15% is obtained, which clearly shows that there is no deviation from normality. The Normal QQ plot in Fig. 7a gives the same results as the Kolmogorov–Smirnov test.

A diagnostic analysis of residuals is conducted to investigate the assumptions of the regression model. Figure 7b shows residual vs fitted plot for checking the equality of the error variances. This plot does not show any pattern of heteroscedasticity. A Durbin–Watson test is used to test the correlation among the residuals produced a test statistic of d = 1.63, and the 5% significance levels of the upper and the lower critical values are dL, 0.05 = 1.62 and dU, 0.05 = 1.71, respectively. Since 4–d is more than dU, 0.05, the test supports the claim that errors are not correlated.

**Figure 8:**
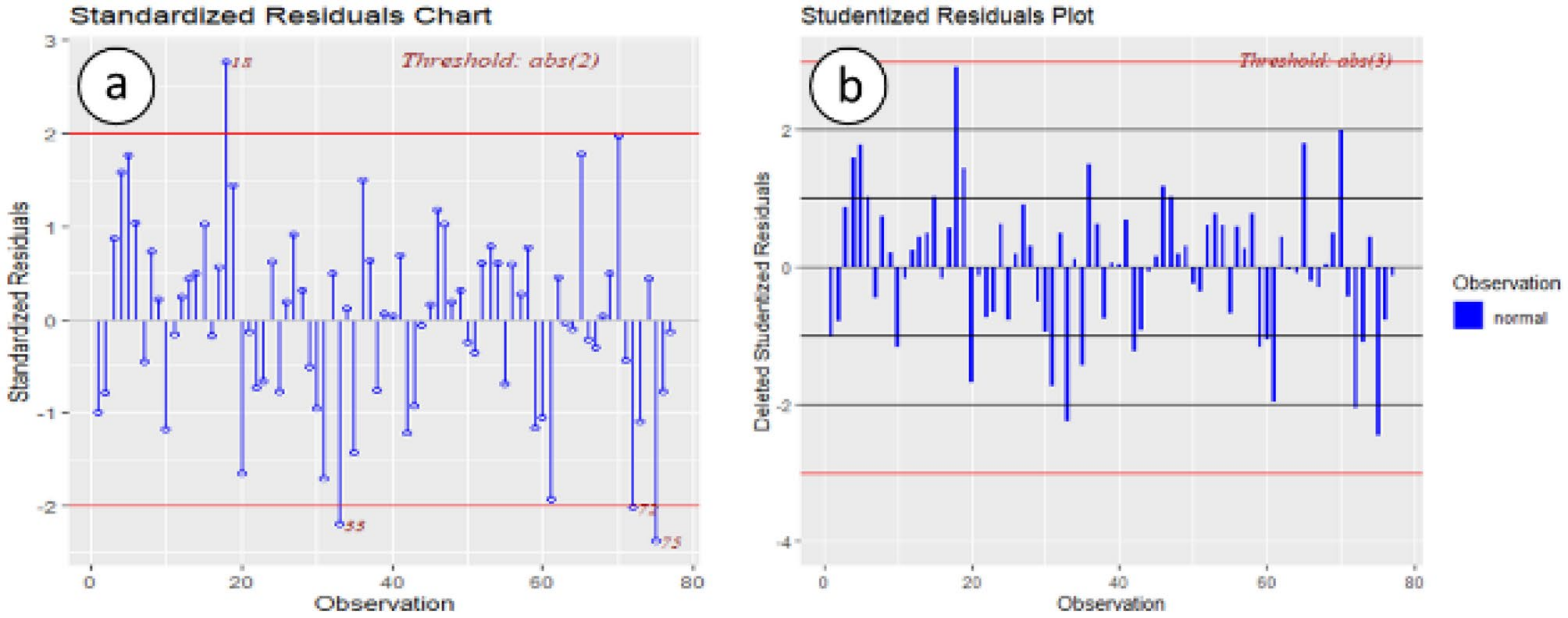
(**a**) Standardized residuals, (**b**) studentized residuals.

Besides, based on the studentized residuals plot in Fig. 8b, all the residuals are in the normal range, but the plot of the standardized residuals in Fig. 8a shows that there are three outliers. After the investigation of these outliers, it is realized that those are genuine values caused by data variation as usual.

### Adaptive neuro fuzzy inference system (ANFIS)

**Figure 9:**
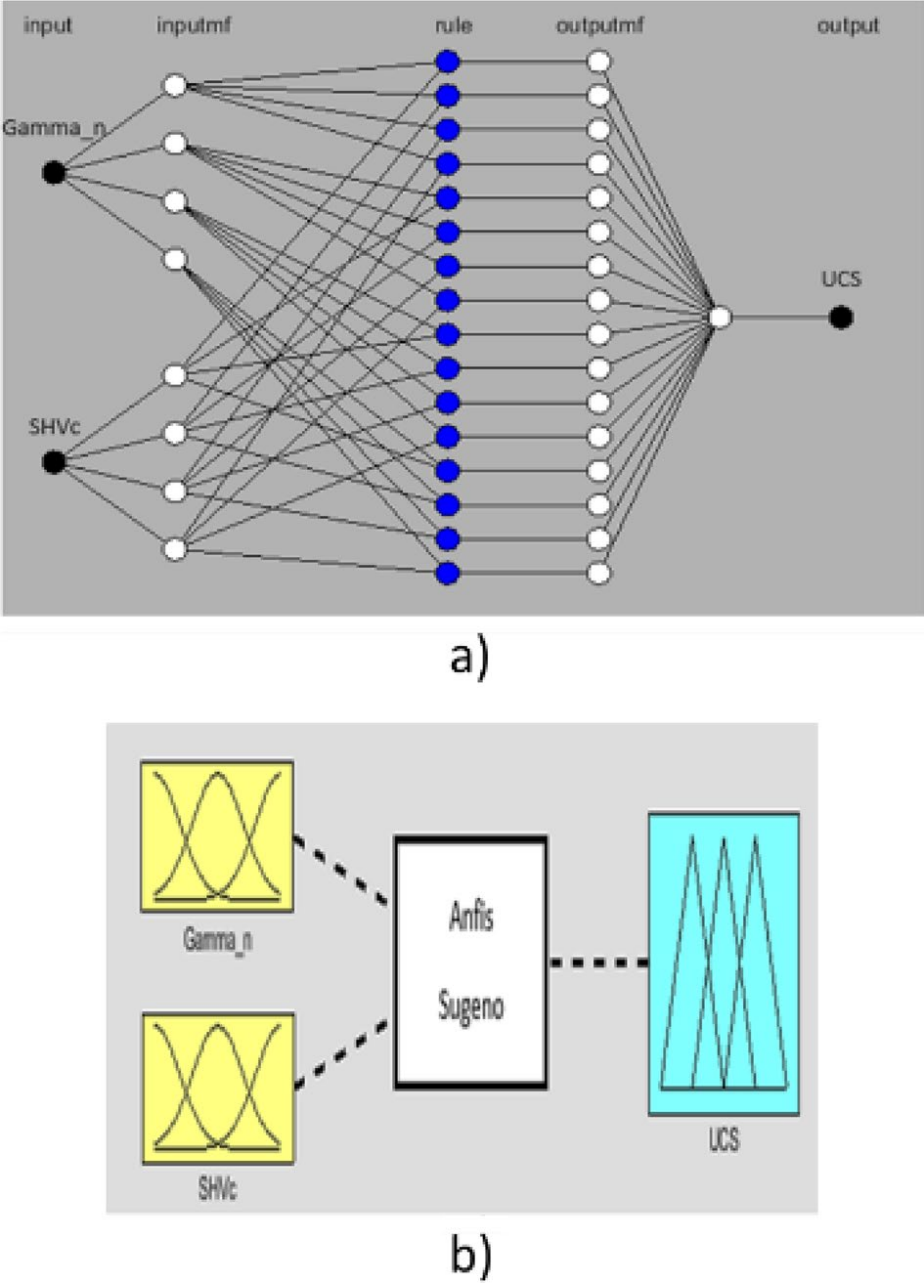
(**a**) The Anfis fitted model, (**b**) the Anfis path.

In this section of the study, the best sugeyno-type Anfis model for the prediction of the UCS values is investigated using the two explanatory variables γ_n_ and SHVc as shown in Fig. 9b. The best model chosen by the accuracy measures, the MAE, RMSE and the R^2^, is the model with eight Gaussian membership functions, four for each input variable, and a total of 16 rules as presented on Fig. 9a. The accuracy measures produced by the model are listed in Table 3.

**Table 3:** Performance comparison of the models.

Model	R^2^	MAE	RMSE
SANN	0.450	17.91	24.15
PCR	0.402	19.15	25.92
MLR	0.402	18.89	25.99
ANFIS	0.425	19.26	26.34

The overall results of the accuracy measures for the prediction of the UCS values from the four competing models are given in Table 3. These results indicate that there are no significant differences among the three models. However, based on all accuracy measures, RMSE and MAE and R^2^, SANN has a slight advantage against the other three models.

**Figure 10:**
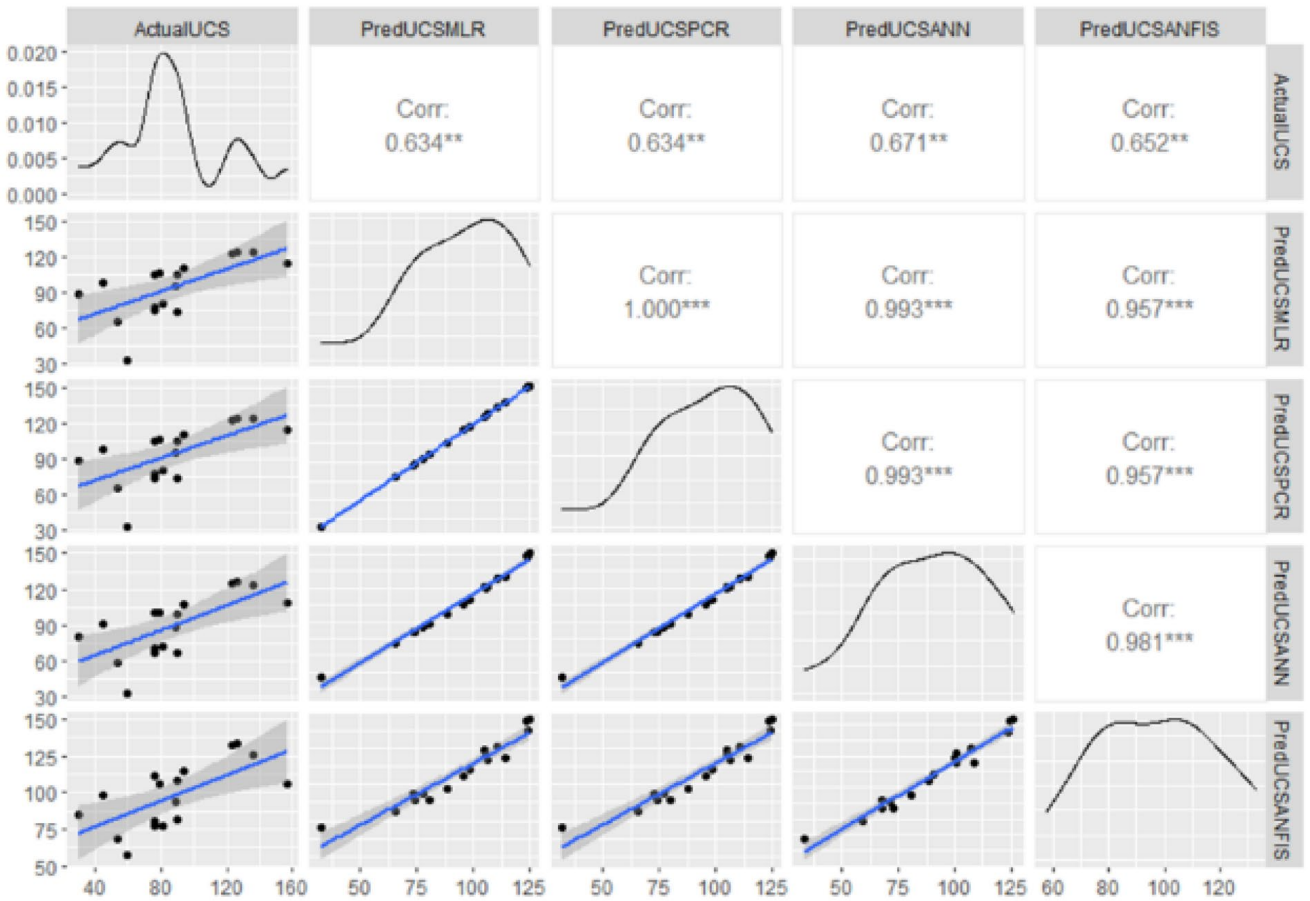
Estimated regression models for the three techniques.

Figure 10 shows the performance of the compared models. The correlations between the actual UCS and each of the predicted values from the four models, MLR, PCR, SANN and ANFIS are 0.634, 0.634, 0.671, respectively.

**Figure 11:**
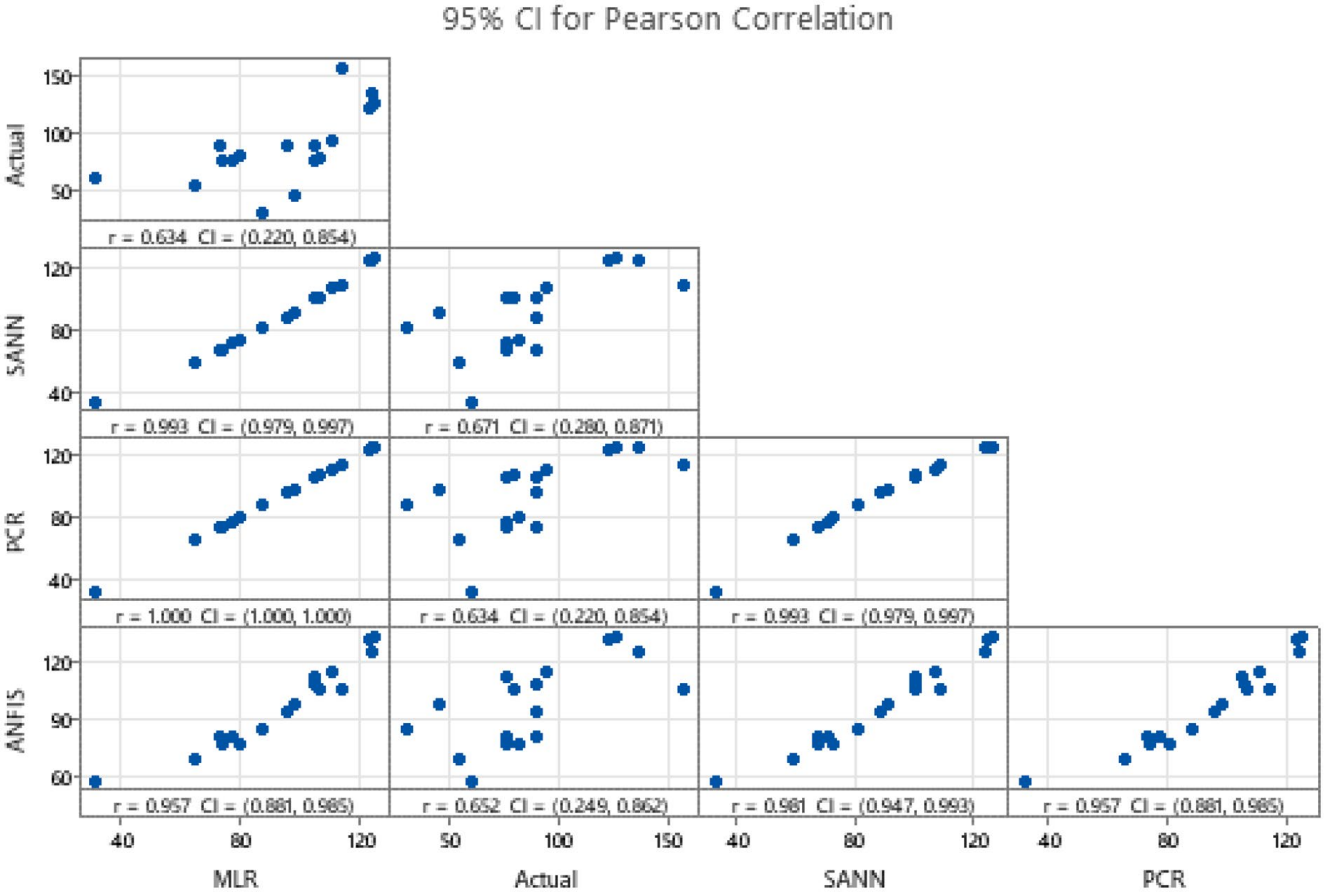
95% percent confidence interval for the correlations of the actual and the predicted values.

The 95% confidence intervals for the correlations of the actual and the predicted values are presented in Fig. 11. Those results indicate the importance of the explanatory variables in predicting UCS (**means P < 0.01, and ***P < 001).

**Figure 12:**
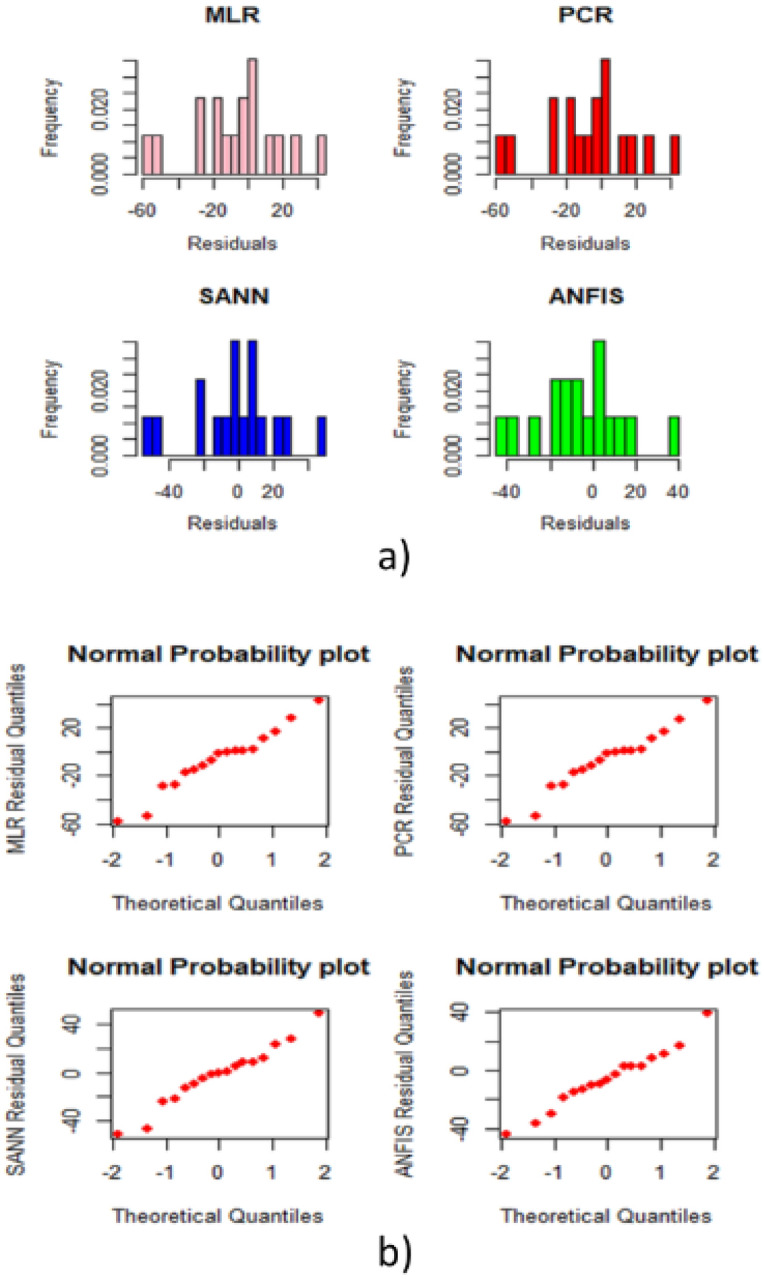
(**a**) Residual histograms, (**b**) residual QQplots.

Qualitative analysis of the residuals from the prediction results of the four models are presented on the histograms in Fig. 12a and the normal QQplots on Fig. 12b. Both plots have shown that the residuals are not deviated from Normality. In addition to those plots, a Kolmogorov–Smirnov goodness of fit tests are conducted, and the results obtained from these tests are all significant with p values of more than 0.15, which is clearly supporting the outcome of the qualitative analysis.

## Conclusions

In this study, the prediction performance of four machine learning techniques, principal components regression, multiple least squares regression, sequential artificial neural networks, and adaptive neuro fuzzy inference system, are compared to predict UCS values using Schmidt Hammer (SHV_C_) test on core sample and a unit weight (γ_n_) of carbonate rock as explanatory variables. Those techniques have a history of high-quality estimation as they provide highly accurate predictions. The findings of this study are as follows:

1. Results of the study have demonstrated that all the methods are useful and competitive, but based on both RMSE and MAE; SANN has a slight advantage against the other three models.
2. The limitation of the study is data variation caused by outliers, which is a common problem for real data, those variations have negatively affected prediction accuracy measures, but the four employed models fitted the data very well.
3. Correlations among the predictions have also shown close performances of the different models. As can be seen from Fig. 10, PredUCSPCR, and PredUCSANN is 0.996, whereas the correlation between PredUCSPCR and PredUCSMLR is 0.995, the correlation between PredUCSMLR and PredUCSANN is 0.993.

Finally, the correlations of PredUCSANFIS with the other three predictions, PredUCSPCR, PredUCSANN and PredUCSMLR are 0.959, 0.980 and 0.957, respectively. The R^2^ values for these regressions are 99.2%, 99%, and 98.6%, 92%, 96% and 92% correspondingly.

## Data Availability

The datasets generated during and/or analyzed during the current study are available from the corresponding authors on reasonable request.
